# Hygienic quality of soil in the Gemer region (Slovakia) and the impact of risk elements contamination on cultivated agricultural products

**DOI:** 10.1038/s41598-021-93587-w

**Published:** 2021-07-08

**Authors:** Janette Musilová, Ľuboš Harangozo, Hana Franková, Judita Lidiková, Alena Vollmannová, Tomáš Tóth

**Affiliations:** Department of Chemistry, Faculty of Biotechnology and Food Sciences, SUA Nitra, Tr. A. Hlinku 2, 949 76 Nitra, Slovakia

**Keywords:** Agroecology, Ecology

## Abstract

The Gemer region (Slovakia) belongs to areas with a high concentration of risk elements. The contents of Cu, Ni, Pb, Cd, Hg and Mn were determined in soils and cultivated agricultural production from lands in three cadastres of the Gemer region (Henckovce (48.713845, 20.426189) and Nižná Slaná (48.717373, 20.4208423), cultivated crop—spring triticale; Gemerská Poloma (48.704523, 20.487645), cultivated crop—maize). Bioavailable forms of Pb (0.306–0.532 mg/kg) and Cd (0.104–0.154 mg/kg), pseudototal forms of Cd (3.07–3.98 mg/kg) and Hg (0.484–2.18 mg/kg) represented the highest risk in soils. The highest contents of Pb (Cd, Hg) were in maize from Gemerská Poloma: 0.898 (0.081, 0.399) mg/kg DM. Soils were classified based on several indicators of soil contamination. The highest values of indicators are: Contamination factor (C_f_ 29.1—Hg), Degree of contamination (C_deg_ 51.5), Potential ecological risk factor (E_r_ 1163—Hg), Potential ecological risk index (RI 1,520), Pollution load index (PLI 4.76), Geo-accumulation index (I_geo_ 5.60—Hg). All indicators concerned the lands of Henckovce and Gemerská Poloma, RI also concerned the land of Nižná Slaná. Bioaccumulation factor (BAF) was calculated to assess the plant's ability to absorb the risk element. For both crops and all risk elements, BAF values were < 1. Obtained results indicate heavy metal contamination of soils, therefore monitoring of soils in investigated area is necessary.

## Introduction

Soils are a key of enabling resource, central to the creation of a host of goods and services integral to ecosystems and human well-being. In agriculture and forestry, they are considered as a basic means of production and an important natural resource as well as a part of the environment and the wealth of society^1,2^.

In general, geogenic (natural) and anthropogenic activities are a responsible sources of soil contamination by heavy metals^3^.

The main natural sources of heavy metals are atmospheric deposition (dust blown by the wind), as well as volcanic activity, forest fires, vegetation, sea salt, etc^3,4^.

Principal anthropogenic sources include industrial emissions, mining, smelting^5^, but also agriculture (wastewater irrigation, application of fertilizers and pesticides)^3^, urban activities (fuel combustion processes and transport, urbanisation, industrialisation, sewage water)^4,6–8^.

Numerous studies have shown that sources of heavy metal pollution in the environment come mainly from anthropogenic sources. Mining is considered to be one of the most important anthropogenic sources of heavy metal contamination^6^. Mining industry causes environmental degradation (soil and water contamination), deforestation and desert formation, floods, landslides and storms^9^. Soil pollution by heavy metals has become a serious problem in many parts of the world. Old environmental burdens and discontinued mining activities are significant sources of environmental contamination.

As part of a systematic identification, 257 environmental burdens were registered in Slovakia. There are almost 17,000 old mining works, including tunnels (4873), shafts (517), heaps (6.125), sludge ponds (10) and other works, which are located in most regions. The regions most affected by the old mining activity include Spiš (copper, silver, gold, mercury, lead and iron ore), Hont (silver, ore processing), the Upper Nitra region (brown coal mining) and Gemer (iron ore, silver, gold, copper, pure mercury, cinnabarite, cobalt–nickel ores)^10,11^.

Due to its natural mineral resources, Gemer was in the past one of the most developed industrial areas at that time in then Austria-Hungary. This development was related to ore mining. In the nineteenth century, the area of Gemer had the largest concentration of mines in Hungary. In addition to mining, metallurgy and glassmaking (Gemer and Malohont) was developed in the northern areas (near to Dobšiná and on the upper course of the Slaná River). The first iron ore mines were established in Nižná Slaná 800 years ago. In 1975, a plant for the processing of siderite ores “Siderit” was established. The main mass of the deposit in Nižná Slaná consisted of metasomatic ankerite and siderite, which carry a substantial part of iron. Fine-grained dark grey siderite is highly ferrous and also has an increased Mn content^12^. More than 700-year mining and processing of siderite ores was completed in 2008^2^. After the end of ore mining and processing, a tailings pond (465 m a.s.l.) remained in the forests above the village, which is still an environmental burden for the population. Almost 7 million cubic meters of the sewage sludge are stored on the tailing pond in Nižná Slaná, covering a total area of 20.6 ha^13^. Sludge has the consistency of the fine-grained material that is, during the windy weather, transmitted to the surrounding villages. Due to the unclear ownership relations, the lack of reclamation and the rehabilitation of the dam, security, and stability of the tailing pond is questionable^14^. The whole area is currently included in the list of environmental burdens^15^.

The main objective of this study was to assess the risk of soil contamination by heavy metals, which is a consequence of the old environmental burden in the northern part of the Gemer region. The degree of contamination was expressed on the basis of the value of the Contamination factor (C_f_), the Degree of contamination (C_deg_), the Potential ecological risk factor (E_r_), the Potential ecological risk index (RI), the Pollution load index (PLI) and the Geo-accumulation index (I_geo_). In addition, the aim was to assess the risk of the monitored heavy metals entering crops grown on such contaminated agricultural land using the Bioaccumulation Factor (BAF).

## Material and methods

Sampling points are located in three cadastres of the Rožňava district in the Gemer region. In the past, this area was characterized by iron ore mining and the processing of metasomatic siderites had a tradition of more than 600 years. The siderite base consists of siderite deposits in Nižná Slaná, which are part of the Gelnica series in the Spiš-Gemer Ore Mountains^2^. Mining is currently suspended.

Arable land in the Rožňava district makes up 8.9% (10.481 ha) of the total area^16^. The predominant soil types of agricultural land are fluvisol (12.64%) and cambisol (52.70%)^17^.

### Sampling and sample preparation

Experimental research and field studies on plants, complies with relevant institutional, national, and international guidelines and legislation (Decree 151/2016^51^). Soil samples and samples of plant material were collected from three plots with an area of 12.8 ha (cadastre of Gemerská Poloma (48.704523, 20.487645), cultivated crop – maize), 12.1 ha (cadastre of Henckovce (48.713845, 20.426189), cultivated crop—spring triticale) and 23.41 ha (cadastre of Nižná Slaná (48.717373, 20.4208423), cultivated crop—spring triticale). 5 average samples of soil and plant material were collected from each plot. The sampling method was as follows: five sampling points (A, B, C, D, E) with an area of approx. 10 × 10 m were determined in advance on the plot (four samples were taken from the corners of each plot and the fifth from the center). In this area, 5 sampling points were selected in a similar way, from which soil samples at the horizont 0–0.1 m into the pedological probe GeoSampler by Fisher (approx. 1 kg from one sampling point) and plant samples (approx. 0.5 kg from one sampling point) were taken. One average sample was prepared from each sampling site (A – E). Subsequently, the samples were dried. Soil samples were ground (grinding machine VEB Thurm ZG 1) to *fine earth I* (average particle size 2 mm), in which the agrochemical characteristics (pH/KCl, were determined. Content of risk elements was determined in *fine earth II* (average particle size 0.125 mm). Only purified grain from plant samples was used for analysis, which was pulverized (Grindomix 200 GD; Retsch, Germany). The content risk elements in plant samples was determined.

### Chemical analysis

The exchange soil reaction (pH/KCl), which is given by the change in hydrogen ion activity, was determined potentiometrically (691 pH Meter Metrohm, Herisau, Switzerland) in KCl suspension (20 g *fine earth I* + 50 mL KCl, c = 1 mol/L; KCl: CentralChem, Slovakia) after 10 min of extraction at a frequency of 180 oscillations/min (Unimax 2010 horizontal shaker; GmbH, Germany).

The contents of two forms of risk elements (Cu, Ni, Pb, Cd, Mn) were determined using Flame AAS method (Cu, Ni, Mn) and Graphite Furnace AAS method (Cd, Pb). The mobile forms were determined in soil extract by NH_4_NO_3_ (c = 1 mol/L; NH_4_NO_3_; Merck, Germany). The total contents of risk elements, including all metal forms with exception of silicate forms, were determined in soil extract by *aqua regia* (1 g *fine earth II* + 10 mL *aqua regia*, HNO_3_, HCl; Merck, Germany) after microwave digestion (70 min, MARS X-Press 5, CEM Corp., Matthews, NC, USA). Cu (Ni, Pb, Cd, Mn, Hg) were determined at wavelength 324.8 (232, 217, 228.8, 279.5, 253.65, resp.) nm, Limits of detection LOD were 0.1 (0.4, 1.0, 0.05, 0.15, 1.5, resp.) mg/kg and Limits of quantification LOQ were 0.3 (1.2, 3.0, 0.15, 0.45, 4.5, resp.) mg/kg.

The total Hg content was determined using the AAS method with cold Hg vapour detection (AMA 254, Altec, Prague, CZE).

The contents of risk elements (Cu, Ni, Pb, Cd, Mn) in the plant material was determined after their mineralization using a closed microwave digestion system (Mars X-Press 5) without the use of hydrogen peroxide by F-AAS method (Cu, Ni, Mn) and GF-AAS method (Cd, Pb).

The measured results were compared with multi-elemental standard for GF-AAS (Merck, Germany).

The contents of risk elements determined in soil samples were compared with limit and critical values according to Act No. 220/2004^18^. The contents of risk elements determined in plant samples were evaluated according to maximum allowed amounts given by Government Decree 438/2006^19^.

### Indicators of soil contamination by risk elements

1. Contamination factor (C_f_) is given by the ratio of the concentration of a given element in the soil and its background concentration (Eq. 1) (hazardous elements in the pre-industrial soil of the study area)^20^:
  1 $${\mathrm{C}}_{\mathrm{f}}^{\mathrm{i}}=\frac{{\mathrm{C}}^{\mathrm{i}}}{{\mathrm{B}}^{\mathrm{i}}}$$
  where: ${\mathrm{C}}_{\mathrm{f}}^{\mathrm{i}}$ is the single element contamination factor, ${\mathrm{C}}^{\mathrm{i}}$ is the determined concentration of the risk element, ${\mathrm{B}}^{\mathrm{i}}$– level of geochemical background.
  Background values according to Linkeš et al.^21^ were used to calculate ${\mathrm{C}}_{\mathrm{f}}^{\mathrm{i}}$. For manganese, the value of ${\mathrm{B}}^{\mathrm{i}}$ is not given in this publication, so the value according to He et al.^22^ was used.
2. Degree of contamination (C_deg_) is the sum of contamination factors for all risk elements examined (Eq. 2):
  2 $${\mathrm{C}}_{\mathrm{d}\mathrm{e}\mathrm{g}}=\,\,\sum \frac{{\mathrm{C}}^{\mathrm{i}}}{{\mathrm{B}}^{\mathrm{i}}}=\,\,\sum {\mathrm{C}}_{\mathrm{f}}^{\mathrm{i}}$$
  The sum of ${\mathrm{C}}_{\mathrm{f}}^{\mathrm{i}}$ for all metals represent the integrated pollution degree of the environment^20^.
3. Potential ecological risk factor (E_r_) is used to assess the toxicity of trace elements. Its calculation is based on the Eq. (3):
  3 $${\mathrm{E}}_{\mathrm{r}}^{\mathrm{i}}=\,\,{\mathrm{T}}_{\mathrm{r}}^{\mathrm{i}\,\,}\times \,\,{\mathrm{C}}_{\mathrm{f}}^{\mathrm{i}}$$
  where: ${\mathrm{E}}_{\mathrm{r}}^{\mathrm{i}}$ is the potential ecological risk factor – calculated for individual risk element (single risk factor of risk element)^23^, ${\mathrm{T}}_{\mathrm{r}}^{\mathrm{i}}$ is the represents the toxicity index of each element, whose standard value are: Mn = 1, Cu = Ni = Pb = 5, Cd = 30, Hg = 40^23,24^.
4. Potential ecological risk index (RI) represents the toxicity of all studied risk elements and is calculated as the sum of ${\mathrm{E}}_{\mathrm{r}}^{\mathrm{i}}$ (Eq. 4):
  4 $$\mathrm{R}\mathrm{I}\,\,=\,\,\sum{\mathrm{T}}_{\mathrm{r}}^{\mathrm{i}}\times\,\,{\mathrm{C}}_{\mathrm{f}}^{\mathrm{i}}\,\,=\,\,\sum{\mathrm{E}}_{\mathrm{r}}^{\mathrm{i}}$$
5. Pollution load index is another indicator of soil quality in terms of its contamination by risk elements. The Pollution load index (PLI) is defined as the n-th root of the multiplications of the contamination factor (${\mathrm{C}}_{\mathrm{f}}^{\mathrm{i}}$) of metals^20^. The Eq. (5) for calculating PLI is:
  5 $$\mathrm{P}\mathrm{L}\mathrm{I}={(\mathrm{C}}_{\mathrm{f}1}^{\mathrm{i}}\times \,\,{\mathrm{C}}_{\mathrm{f}2}^{\mathrm{i}}\times \,\,{\mathrm{C}}_{\mathrm{f}3}^{\mathrm{i}}\times \dots \,\,\times \,\,{\mathrm{C}}_{\mathrm{f}\mathrm{n}}^{\mathrm{i}}{)}^{\frac{1}{\mathrm{n}}}$$
6. The Geo-accumulation index enables the assessment of environmental contamination differences between current and pre-industrial concentrations^6,25^. Geo-accumulation index (I_geo_) is calculated according to the Eq. (6):
  6 $${\mathrm{I}}_{\mathrm{g}\mathrm{e}\mathrm{o}}=\,\,{\mathrm{l}\mathrm{o}\mathrm{g}}_{2}\left(\frac{{\mathrm{C}}^{\mathrm{i}}}{\mathrm{1,5}\times {\mathrm{B}}^{\mathrm{i}}}\right)$$
  where: 1.5 is the is the constant, which was used to correct possible variations in the background values of a particular metal in the environment^26,27^.
7. Transfer of risk elements from soil to plant is expressed by Bioaccumulation factor. Its value is calculated from the ratio of the concentration of risk element in the plant ${\mathrm{C}}_{\mathrm{p}}^{\mathrm{i}}$ and in the soil ${\mathrm{C}}_{\mathrm{s}}^{\mathrm{i}}$ ^5,28^ (Eq. 7):
  7 $${\mathrm{B}\mathrm{A}\mathrm{F}}_{i}=\,\,\frac{{\mathrm{C}}_{\mathrm{p}}^{\mathrm{i}}}{{\mathrm{C}}_{\mathrm{s}}^{\mathrm{i}}}$$

### Statistical analyses

Results were evaluated using descriptive statistical analysis (Microsoft Excel, Redmond, WA, USA) and analysis of variance (ANOVA, multi-range tests, method: 95.0 percent LSD) using Statgraphics statistical software (Centurion XVI.I, USA).

### Declaration

Experimental research and field studies on plants, complies with relevant national guidelines and legislation (Decree of the Ministry of Agriculture and Rural Development of the Slovak Republic, which establishes the details of agrochemical testing of soils and storage and use of fertilizers 151/2016).

## Results and discussion

### Soil

#### Contents of risk metals in soils

Lands of localities from which soil and plant samples were taken belong to agricultural lands.

Soil reaction is one of the factors that most affects the behaviour of heavy metals in soil. Low pH values pose a risk of reduced nutrient intake and increase the availability of heavy metals for plants^29,30^.

The presence of risk elements in the soil was evaluated based on their contents in bioavailable form (mobile forms), determined in soil extracts NH_4_NO_3_, and the total contents of risk elements were determined in soil extract by *aqua regia* (Table 1).

**Table 1:** The contents of risk elements (Cu, Ni, Pb, Cd, Hg, Mn) in soil (mg/kg).

	Cu	Ni	Pb	Cd	Mn	Hg
**Cadastre**	**Bioavailable forms**					
**Henckovce**	0.054–0.079	0.170–0.206	0.257–0.330	0.087–0.114	2.34–4.66	
Mean ± SD	0.064 ± 0.011^a^	0.191 ± 0.015^a^	0.306 ± 0.029^a^	0.106 ± 0.011^a^	3.47 ± 0.911^a^	
**Nižná Slaná**	0.039–0.066	0.253–0.345	0.265–0.370	0.095–0.118	11.90–22.80	
Mean ± SD	0.051 ± 0.010^a^	0.290 ± 0.037^b^	0.310 ± 0.038^a^	0.104 ± 0.009^a^	16.2 ± 4.70^b^	
**Gemerská Poloma**	0.108–0.390	0.255–0.350	0.503–0.677	0.133–0.195	0.72–3.97	
Mean ± SD	0.172 ± 0.123^b^	0.302 ± 0.041^b^	0.532 ± 0.087^b^	0.154 ± 0.026^b^	2.12 ± 1.35^a^	
Critical value^18^	1.00	1.50	0.10	0.10	–	
**Cadastre**	**Pseudototal forms**					
**Henckovce**	24.7–30.9	30.4–39.8	34.5–39.2	3.16–4.30	1,101–1,509	1.16–2.29
Mean ± SD	28.1 ± 2.75^a^	34.7 ± 3.78^a^	37.3 ± 1.84^a^	3.71 ± 0.438^b^	1,294 ± 165^a^	1.74 ± 0.533^ab^
**Nižná Slaná**	21.8–26.0	31.8–35.0	41.2–45.7	3.48–4.37	911–1216	0.369–0.676
Mean ± SD	23.7 ± 1.55^a^	33.2 ± 1.23^a^	42.5 ± 1.85^b^	3.93 ± 0.345^b^	1,060 ± 119^a^	0.484 ± 0.148^a^
**Gemerská Poloma**	19.1–211	24.2–45.7	38.0–44.2	2.51–3.28	561–1364	0.235–5.47
Mean ± SD	98 ± 79.7^b^	37.4 ± 10.8^a^	41.5 ± 2.51^b^	3.07 ± 0.317^a^	1100 ± 345^a^	2.18 ± 2.03^b^
Limit value^18^	60.0	50.0	70.0	0.70	–	0.50

^a,b^Statistically significant differences between cadastres, P value < 0.05.

Accessible heavy metals for plants are those which are present in the soil solution as soluble components or those which are easily dissolved by root exudates^31^. The highest Cu contents determined in soil extract by NH_4_NO_3_, were in the cadastre of Gemerská Poloma (max. 0.390 mg/kg) (Table 1). However, even the highest determined concentration of Cu in its bioavailable form did not exceeded the determined critical value for this element^18^. Nickel is a beneficial element for plants. Elevated Ni concentrations in soils have a potential negative effect on plants^32^. Content of bioavailable forms of nickel is lower than the determined critical value in all analysed samples. Cadmium and lead present a risk to agricultural activity in this area. Cadmium in soil is highly bioavailable and has higher mobility in plants compared to other heavy metals. It is easily transported by roots to shoots. In contrast, lead is one of the least mobile heavy metals. It is naturally concentrated in the upper layers of the soil^33^. The contents of the available forms of cadmium and lead exceed the critical values for these elements. In case of lead, the determined contents are from 0.257 Henckovce to 0.676 Gemerská Poloma. Takáč et al.^34^ determined in 20 soil samples from the Central Spiš region 7.2–257.6 mg Cu/kg soil and 1.0–84.8 mg Pb/kg in their potentially mobilizable form and 0.4–1.4 mg Cu/kg soil and 4.3–7.1 mg Pb/kg in their mobile form. In comparison with our results, Vilček et al.^35^ determined a lower content of Cd (0.04), Pb (0.17), Ni (0.15) and higher Cu content (0.48) mg/kg in forms accessible to plants in 16 soil samples from locality Nižná Slaná in the years 2006–2008. However, high concentrations of metals in soil do not necessarily mean the availability of metals for plants^36^. As a result, extractable Mn is often a better indicator of Mn availability. Mn^2+^ is generally considered to be bioavailable^22^. The highest concentration of Mn was measured in soil samples from the cadastre of Nižná Slaná. On the contrary, the lowest concentrations were detected in samples from Gemerská Poloma cadastre, which is the furthest cadastre from the source. No critical limit is set up for manganese according to Slovak legislation, it is not possible to classify these soils as contaminated/uncontaminated. For comparison, the EDTA-extractable content of Mn ranged from 22.7 to 127 mg/kg dry soil (China)^29^; the mobile concentrations between 0.32 and 202.0 mg/kg and the available concentrations from 5.4 to 126.3 mg/kg (Egypt)^37^.

Based on results of statistical analysis, significant higher content of Cu, Pb and Cd can be stated in samples from Gemerská Poloma cadastre. These soils are classified as gley fluvisols, soils from the other two localities are cambisols (from medium heavy to light) and acid cambisols (Henckovce), cambisols from medium heavy to light and typically acid cambisols (Nižná Slaná). The soil profile of fluvisols is repeatedly disrupted by floods, which often enriches them with a new layer of sludge sediments^2^.

Another method for determination of metal content in soil is mineralisation using *aqua regia*, which dissolves most of the soil constituents except those strongly bound in silicate minerals. This content is sometimes referred to as pseudototal (determined in *aqua regia*). In this way, all elements that are likely to become bioavailable in the long term are determined^38^.

Pseudototal contents of risk metals (Table 1) determined in soil extract using *aqua regia* were higher than their limit value in case of Cu (Gemerská Poloma cadastre), Cd (all cadastres) and Hg (cadastre of Henckovce and Gemerská Poloma).

Due to the fact that the hygienic condition of agricultural soils is assessed according to the exceeding of the limit values of at least one risk substance, the monitored plots can be classified as contaminated (Cu > 60.0, Cd > 0.7, Hg > 0.5 mg/kg soil).

Manganese is not classified as risk element in Slovak legislation.

Tóth et al.^39^ classified European soils into four categories: (1) no detectable content of HM, (2) the concentration of the investigated element is above the threshold value (Hg 0.5, Cd 1, Cu 100, Pb 60 and Ni 50 mg/kg), but below the lower guideline value (Hg 2, Cd 10, Cu 150, Pb 200 and Ni 100 mg/kg), (3) concentration of one or more element exceeds the lower guideline value but is below the higher guideline value (Hg 5, Cd 20, Cu 200, Pb 750 and Ni 150 mg/kg), (4) samples having concentrations above the higher guideline value.

In comparison with the threshold and guideline values, soils in cadastres of Gemerská Poloma (Cu), Henckovce, Nižná Slaná, Gemerská Poloma (Cd, Hg) represent the ecological risk. Threshold and guideline values for Mn were not defined.

The Spiš region and the northern part of the Gemer region belong to the most polluted areas in Slovakia in terms of soil contamination due to mining and metallurgical activities that have been carried out here in the past. Soils near the sludge in Nižná Slaná contain 3.17–53.3 (14.2–301, 0.71–20.6, 3.33–177, 12.9–223 and 675–11,510, respectively) mg Cd (Cu, Hg, Ni, Pb and Mn, respectively)/kg of soil^14^. In loaded area of Dongchuan, (China), contained Cd (Cu, Hg, Ni and Pb, resp.) 0.20–3.57 (45.38–2026, 0.02–0.23, 24.06–95.9 and 6.83–146.6, resp.) mg/kg^40^. In contrast, in the agricultural area of Punjab of the India, the soil contamination was caused by an excessive use of agrochemicals and polluted irrigation sources. Increased Cu (Pb and Cd) contents were determined in the soil samples: 9.0–48.5 (5.5–9.67 and 0.516–1.58, resp.) mg/kg^41^.

However, in most cases, a large portion of the total element content is not available for immediate uptake by plants. Available forms represent a small proportion of this total content which is potentially available to plants. Availability is affected by many factors, including pH, redox state, macronutrient levels, available water content and temperature^29,33,36,38^.

### Indicators of soil contamination

#### Contamination factors and degree of contamination

The contamination character may be described in a uniform, adequate and standardised way by means of the contamination factor and the degree of contamination. Hakanson^24^ reported four Contamination degrees of individual metal (${\mathrm{C}}_{\mathrm{f}}^{\mathrm{i}}$) – low (${\mathrm{C}}_{\mathrm{f}}^{\mathrm{i}}$ < 1), moderate (1 ≤ ${\mathrm{C}}_{\mathrm{f}}^{\mathrm{i}}$  < 3), considerable (3 ≤ ${\mathrm{C}}_{\mathrm{f}}^{\mathrm{i}}$  < 6) and very high (${\mathrm{C}}_{\mathrm{f}}^{\mathrm{i}}$ ≥ 6) and four Contamination degrees of the environment (C_deg_) – low contamination (C_deg_ < 8), moderate contamination (8 ≤ C_deg_ < 16), Considerable contamination (16 ≤ C_deg_ < 32) and Very high contamination indicating serious anthropogenic pollution (C_deg_ ≥ 32).

Average C_f_ values for risk metals (Cu, Co, Ni, Pb, Cd, Hg, and Mn) range from 1.05 (Cu, Nižná Slaná) to 29.1 (Hg, Gemerská Poloma) (Table 2).

**Table 2:** Contamination factor (C_f_) and degree of contamination (C_deg_).

Cadastre	C_f_						C_deg_
	Cu	Ni	Pb	Cd	Hg	Mn	
**Henckovce**							
Mean ± SD	1.25 ± 0.122	2.71 ± 0.295	1.50 ± 0.074	13.0 ± 1.54	23.2 ± 7.11	3.23 ± 0.411	44.9 ± 9.55
Range	1.09–1.37	2.38–3.11	1.39–1.58	11.1–15.1	15.5–30.6	2.75–3.77	34.2–55.5
**Nižná Slaná**							
Mean ± SD	1.05 ± 0.069	2.60 ± 0.096	1.71 ± 0.074	13.8 ± 1.21	6.45 ± 1.98	2.65 ± 0.297	28.3 ± 3.73
Range	0.965–1.15	2.49–2.74	1.66–1.84	12.2–15.3	4.92–9.02	2.28–3.04	24.5–33.1
**Gemerská Poloma**							
Mean ± SD	4.34 ± 3.528	2.92 ± 0.844	1.67 ± 0.101	10.8 ± 1.11	29.1 ± 27.1	2.75 ± 0.863	51.5 ± 33.6
Range	0.845–9.32	1.89–3.57	1.53–1.78	8.81–11.5	3.13–73.0	1.40–3.41	17.6–103
B^i^	22.595	12.79	24.87	0.285	0.075	400	

*B*^*i*^ the background values of risk elements in mg/kg.

Based on values of C_f_, soils in the monitored cadastres can be characterised as contaminated. The values of ${\mathrm{C}}_{\mathrm{f}}^{\mathrm{i}}$ < 1 were not present in any plot. Cd and Hg are high-risk elements, which showed very high contamination degree. Approximately the same average C_f_ values of Ni and Pb were recorded on all three plots (Henckovce, Nižná Slaná, Gemerská Poloma). On two plots (Henckovce, Nižná Slaná), similar average C_f_ values of Cd and Mn on the plots of Nižná Slaná and Gemerská Poloma were recorded. On the contrary, there were significant differences between the average C_f_ values of Cu (Henckovce ≈ Nižná Slaná < Gemerská Poloma), Hg (Henckovce ≈ Gemerská Poloma > Nižná Slaná) and Mn (Henckovce > Nižná Slaná ≈ Gemerská Poloma).

Guo et al.^42^ describes Cf as a pollution coefficient for a certain heavy metal, which can reflect the pollution character of the investigated region but can not reveal the ecological effects and hazards. Based on the contamination factor on scale ranging from 1 to 6, Hakanson^24^ and other authors^37,43,44^ report four degrees of soil contamination by individual risk metals: low–moderate–considerable–very high contamination. Islam et al.^20^ classifies the highest degree of contamination as “high”.

Degree of contamination is indicator of degree of soil contamination by all risk elements. The C_deg_ values determined for the monitored plots are given in Table 2. The C_deg_ values determined for individual sampling sites range from 17.6 to 103 (Gemerská Poloma). According to the classification given by Hakanson^24^, on the basis of average C_deg_ values, lands in the cadastres of Henckovce and Gemerská Poloma show very high contamination degree of the environment, indicating serious anthropogenic pollution and land of Nižná Slaná (mean C_deg_ = 28.3) shows a considerable contamination degree of the environment. Some authors^7,20,44^ use a different scale for soil classification than Hakanson^24^.

According to the soil classification by Luo et al.^44^ and Islam et al.^20^, soils on all three plots show a very high contamination degree.

Among monitored elements, cadmium and mercury are the main contributors to soil contamination. Even if only these two elements were considered for determination of degree of contamination, the soils of Henckovce and Gemerská Poloma plots show a very high degree of contamination (ΣC_Cd_ + C_Hg_ = 36.2, ΣC_Cd_ + C_Hg_ = 80.6% C_deg_ and ΣC_Cd_ + C_Hg_ = 39.9, ΣC_Cd_ + C_Hg_ = 78.1% C_deg_, respectively). Mercury represents 51.7% of C_deg_ (Henckovce) and 56.5% of C_deg_ (Gemerská Poloma). Cadmium is the highest risk on the plot of Nižná Slaná: C_Cd_ = 48.8% C_deg_.

#### Potential ecological risk factor and potential ecological risk index

The toxicity of heavy metals and risk elements is assessed by the ecological risk index. Highly toxic risk elements, present in the soil, especially in their accessible forms, can enter the food chain through cultivated production and animals.

Potential ecological risk factor (${\mathrm{E}}_{\mathrm{r}}^{\mathrm{i}}$) is related not only to the concentration of the risk element present in the soil, but also to the toxicity of each element. The toxicity values determined by Hakanson^24^ are used in the calculation of ${\mathrm{E}}_{\mathrm{r}}^{\mathrm{i}}$: Zn = 1, Cr = 2, Cu = Pb = 5, As = 10, Cd = 30 and Hg = 40.

The values of Potential ecological risk factor, determined based on the total content of risk element and toxicity index, are given in Table 3.

**Table 3:** Potential ecological risk factor (${\mathrm{E}}_{\mathrm{r}}^{\mathrm{i}}$); Potential ecological risk index (RI).

Cadastre	${\mathrm{E}}_{\mathrm{r}}^{\mathrm{i}}$						RI
	Cu	Ni	Pb	Cd	Hg	Mn	
**Henckovce**							
Mean ± SD	2.49 ± 0.244	13.5 ± 1.48	7.50 ± 0.370	391 ± 46.1	929 ± 284	2.16 ± 0.274	1345 ± 333
Range	2.19–2.74	11.9–15.6	6.94–7.88	333–453	621–1,224	1.83–2.51	977–1705
**Nižná Slaná**							
Mean ± SD	2.10 ± 0.137	13.0 ± 0.482	8.55 ± 0.371	414 ± 36.3	258 ± 79.1	1.77 ± 0.198	697 ± 117
Range	1.93–2.30	12.4–13.7	8.28–9.19	366–460	197–361	1.52–2.03	587–848
**Gemerská Poloma**							
Mean ± SD	8.69 ± 7.06	14.6 ± 4.22	8.35 ± 0.505	323 ± 33.4	1163 ± 1084	1.83 ± 0.575	1520 ± 1130
Range	1.69–18.6	9.46–17.9	7.64–8.89	264–345	125–2919	0.935–2.27	409–3312

Based on the values reported by Hakanson^24^ and other authors^20,27,43–45^, the soil of all three plots have a low risk of contamination (${\mathrm{E}}_{\mathrm{r}}^{\mathrm{i}}$ < 40) by metals (Cu, Ni, Pb and Mn). The average values of Potential ecological risk factor for these elements range from 1.77 (Mn, Nižná Slaná) to 14.6 (Ni, Gemerská Poloma). In the case of the site Nižná Slaná, soil samples had a high risk of Hg contamination (160 ≤ ${\mathrm{E}}_{\mathrm{r}}^{\mathrm{i}}$  < 320). In case of ${\mathrm{E}}_{\mathrm{r}}^{\mathrm{i}}$ ≥ 320, soils show a very high risk (very great risk). This value was exceeded for cadmium on all three plots and for mercury on the plots of Henckovce and Gemerská Poloma, where the maximum is ${\mathrm{E}}_{\mathrm{r}}^{\mathrm{i}}$ = 2,919.

There are several numerical intervals for the classification of soils based on Potential ecological risk index (RI), e.g., for very high ecological risk is RI ≥ 260^44^ (400^45^, 600^24,43^). However, even when using the assessment with the greatest tolerance (RI ≥ 600^24^), soils of all three plots pose a very high ecological risk of potential contamination. ${\mathrm{E}}_{\mathrm{r}}^{\mathrm{C}\mathrm{d}}$ summed to ${\mathrm{E}}_{\mathrm{r}}^{\mathrm{H}\mathrm{g}}$ present 96% of RI at the land of Nižná Slaná and 98% of RI at Henckovce and Gemerská Poloma lands.

#### Pollution load index and geo-accumulation index

The PLI gives an assessment of the overall toxicity status of the sample and it is a result of the contribution of all monitored hazardous elements^20^. In our case, the concentrations and contamination factors of six elements were determined. To calculate PLI was derived the six roots of the six factors multiplied together. Results are shown in Table 4.

**Table 4:** Pollution load index (PLI), Geo-accumulation index (I_geo_).

Cadastre	PLI	Cadastre	I_geo_					
			Cu	Ni	Pb	Cd	Hg	Mn
**Henckovce**		**Henckovce**						
Minimum	3.53	A	−0.456	0.743	−0.113	2.89	3.38	0.965
Maximum	4.59	B	−0.181	0.948	−0.016	3.07	4.35	1.11
Median	4.24	C	−0.200	0.664	0.071	3.05	4.20	1.22
Average	4.10	D	−0.399	0.826	−0.004	3.33	3.37	0.876
STDEV	0.437	E	−0.133	1.053	0.053	3.22	4.16	1.33
**Nižná Slaná**		**Nižná Slaná**						
Minimum	3.05	A	−0.528	0.867	0.191	3.03	1.74	0.871
Maximum	3.49	B	−0.382	0.782	0.150	3.29	1.71	0.602
Median	3.12	C	−0.480	0.729	0.293	3.15	2.59	1.02
Average	3.19	D	−0.637	0.817	0.164	3.35	1.78	0.871
STDEV	0.181	E	−0.553	0.760	0.143	3.17	2.44	0.704
**Gemerská Poloma**		**Gemerská Poloma**						
Minimum	2.26	A	−0.541	0.335	0.108	2.55	2.95	1.09
Maximum	7.40	B	−0.827	0.498	0.222	2.94	1.06	−0.096
Median	5.65	C	2.64	1.252	0.027	2.91	5.60	1.19
Average	4.81	D	1.74	1.248	0.161	2.90	4.46	0.661
STDEV	2.12	E	1.88	1.207	0.245	2.88	4.04	1.17

The determined PLI values, which inform about the total level of heavy metal pollution, were in range 2.33–7.23. Varol^46^ evaluates soils according to PLI as uncontaminated, when PLI < 1 (no metal pollution) and contaminated, when PLI > 1 (a pollution exists). According to a more detailed classification^7^, soil contamination can be expressed by seven levels of pollution: background level (PLI 0), no pollution (0–1), moderate to no pollution (1–2), moderate pollution (2–3), high to moderate pollution (3–4), high pollution (4–5), extreme pollution (PLI > 5).

According to average values of PLI and the scale provided by authors, the soils of individual plots can be classified as follows: polluted, PLI > 1 (all plots)^46^; high to moderate pollution (Nižná Slaná), high pollution (Henckovce, Gemerská Poloma), while the soils from three sampling points in Gemerská Poloma showed values of PLI above 5, which mean an extreme pollution^7^.

Another indicator of soil contamination is Geo-accumulation index, according to which is soil quality classified by 7 degrees of contamination: 0—no contamination (Igeo < 0), 1—light (0 < Igeo ≤ 1), 2—slightly moderate (1 < Igeo ≤ 2), 3—moderate (2 < Igeo ≤ 3), 4—hlightly heavy (3 < Igeo ≤ 4), 5—heavy (4 < Igeo ≤ 5), 6—extremely heavy contamination (Igeo > 5)^26^. The seven-level classification is also given by other authors^6,25,27,46^.

The values of I_geo_ of risk metals are given in Table 4. Based on the average values of I_geo_, the plot of Henckovce can be classified as uncontaminated with cooper and lead (I_geo_ < 0), lightly contaminated with Ni, Mn (0 < I_geo_ ≤ 1) and slightly heavy contaminated with Cd and Hg (3 < I_geo_ ≤ 4). Nižná Slaná plot is not contaminated with Cu, but is lightly contaminated with Ni, Pb and Mn, slightly moderate contaminated (1 < I_geo_ ≤ 2) with Hg moderate contaminated with Hg and slightly heavy contaminated with Cd. On the Gemerská Poloma plot, the soil is lightly contaminated (Pb, Mn), slightly moderate contaminated (Cu, Ni) to moderate contaminated (Cd) and slightly heavy contaminated (Hg). Among six risk metals studied, I_geo_ of cadmium and mercury had the highest values on all three plots.

## Plant material

### Content of risk metals

Spring triticale was grown on the plots in the cadastre of Henckovce and Nižná Slaná and maize in the cadastre of Gemerská Poloma. Both crops were used as feed. The contents of risk elements determined in these crops are shown in Table 5. There are statistically significant differences between the contents of risk elements in crops from individual cadastres (p < 0.05). The results obtained were compared with the maximum levels for feeding stuffs^19^. Based on the obtained results, it can be stated that the agricultural production grown on all three plots is uncontaminated in terms of the content of monitored risk metals.

**Table 5:** The contents of risk elements (Cu, Ni, Pb, Cd, Hg, Mn) in plants (mg/kg DM).

Cadastre	Risk elements					
	Cu	Ni	Pb	Cd	Hg*	Mn
**Henckovce**						
A	5.51	0.395	BDL	BDL	0.394	21.7
B	4.61	0.202	BDL	BDL	0.106	25.0
C	4.68	0.203	BDL	BDL	0.237	29.7
D	5.28	0.229	BDL	BDL	0.044	21.3
E	4.82	0.201	BDL	BDL	0.221	22.9
Mean ± SD	4.98 ± 0.395^b^	0.386 ± 0.063^a^	NC	NC	0.200 ± 0.135^a^	24.1 ± 3.44^b^
**Nižná Slaná**						
A	6.79	0.998	0.104	BDL	0.055	37.5
B	6.81	0.597	BDL	BDL	0.240	39.0
C	6.40	0.403	0.498	BDL	0.318	30.7
D	5.43	0.599	BDL	BDL	0.022	26.7
E	5.77	0.501	BDL	BDL	0.276	30.8
Mean ± SD	6.24 ± 0.618^c^	0.631 ± 0.154^b^	0.120 ± 0.215^a^	NC	0.182 ± 0.135^a^	32.9 ± 5.15^c^
**Gemerská Poloma**						
A	1.21	0.509	0.585	BDL	0.212	2.95
B	1.49	0.601	0.898	BDL	0.399	3.15
C	1.23	0.695	0.307	BDL	0.023	3.11
D	1.67	0.500	0.299	0.081	0.267	3.34
E	1.4	0.798	0.215	BDL	0.123	2.86
Mean ± SD	1.40 ± 0.191^a^	0.620 ± 0.130^b^	0.461 ± 0.281^b^	0.016 ± 0.036	0.205 ± 0.143^a^	3.08 ± 0.186^a^
Maximum level^19^	–	–	10	1.0	0.1	**–**

*DM* dry matter, *BDL* below detection limit, *NC* not calculated.

*Content of Hg expressed as μg/kg DM.

^a,b,c^Statistically significant differences between cadastres, P value < 0.05.

In the area of the village of S. Francisco de Assis, which is located near The Panasqueira mine in Central Portugal, soil and plant samples were taken from 13 sampling points. Soil samples contained 13.2–36.4 (24.4–134.5, 0.4–2.8 and 168–1,194, respectively) mg/kg Ni (Pb, Cd and Mn, respectively). The contents of Ni (Pb, Cd and Mn, respectively) in plant samples were in the range of 0.3–26.6 (0.2–4.2, 0.1–0.4 and 11.4–270.2, respectively) mg/kg. Cd and Pb concentrations are above the maximum permitted level for vegetables proposed by FAO/WHO (Cd = 0.1 mg/kg, Pb = 0.2 mg/kg, Mn = 500 mg/kg^47^.

Heavy metal contents determined in agricultural soils in the Swat District, northern Pakistan did not represent increased contamination by heavy metals (Cu 0.24–0.61, Ni 0.26–0.77, Cd 0.06–0.14, Mn 0.96–10.05 mg/kg), while the crops contaminations exhibited variations relative to WHO permissible limits. The Cu (Ni, Cd and Mn, respectively) content in maize was 0.19 ± 0.02 (0.28 ± 0.20, 0.11 ± 0.01 and 0.27 ± 0.06, respectively) and in triticum sativum 0.25 ± 0.002 (0.32 ± 0.21, 0.10 ± 0.01 and 1.18 ± 0.17, respectively) mg/kg^3^.

### Indicators of soil contamination—bioaccumulation factor

The ratio of heavy metal concentration in the plant (${\mathrm{C}}_{\mathrm{p}}$) and in the soil (${\mathrm{C}}_{\mathrm{s}}$) is often referred to as Metal Bioaccumulation Factor, The bioaccumulation factor (BAF resp. BF)^28,41^, Transfer factor, resp. Plant transfer factor (TF)^48^, Plant bioaccumulation (PB)^8^, Soil–Plant Transfer Coefficient (TC)^49^.

BAF reflects the plant's ability to absorb heavy metal and is based on the dry weight concentration of HM in plant and soil samples. Usually, the total heavy metal concentration in soil, or the concentration of heavy metal determined in *aqua regia* is considered in its calculation. Table 6 shows BAF values for two crop species (spring triticale and maize) grown on the monitored plots.

**Table 6:** Bioaccumulation factor (BAF).

Cadastre	BAF					
	Cu	Ni	Pb	Cd	Hg	Mn
**Henckovce**						
Minimum	0.154181	0.005050	NC	NC	3.77732E−05	0.015179
Maximum	0.223077	0.012305	NC	NC	3.35457E−04	0.021310
**Nižná Slaná**						
Minimum	0.249083	0.012673	NC	NC	5.69777E−05	0.024326
Maximum	0.288936	0.028514	0.010941	NC	6.50825E−04	0.042834
**Gemerská Poloma**						
Minimum	0.005840	0.010941	0.004864	NC	4.20231E−06	0.002077
Maximum	0.078010	0.022177	0.023632	0.024845	1.69757E−03	0.005702

*NC* not calculated.

BAF values above 1 for heavy metals are considered hazardous to plant and animal health^41,50^. In our study, this value was not exceeded in any case. For spring triticale from both localities (Henckovce, Nižná Slaná) BAF decreased in order Cu > Mn > Ni > Hg > Pb/Cd. In this evaluation, BAF value for Pb (Nižná Slaná) was not considered, because Pb was determined only in samples from two sampling points. For maize (Gemerská Poloma), the highest value of BAF was for Ni and decreased in the order Ni > Cu > Pb > Mn > Hg > Cd. As in the previous case, BAF value for Cd, which was determined in only one sample, was not included in the comparison. Based on the comparison of BAF in individual crops, it can be stated that maize has a higher bioaccumulation capacity of Hg and Pb and lower bioaccumulation of Cu and Mn than spring triticale. In the case of Ni and Cd, the differences are not clear. Other authors^8,27,41^ also report different ability of plants to absorb heavy metals from soil, while their ability is affected by the bioavailability of these metals.

Soil–plant transfer coefficient is an imperative component of human exposure to heavy metals through food chain as it describes movement of contaminants from soil to plants^49^.

## Conclusion

Contamination of soils with risk elements is generally a major problem. Among other factors, old environmental loads, as is the case in the northern part of the Gemer region, represent high levels of ecological danger. Soils in this area are characterised by high concentrations of cadmium, lead, and mercury, either in bioavailable or pseudototal forms. The risk of contamination of these soils is indicated by high values of C_f_, C_deg_ (Hg—Henckovce, Gemerská Poloma), E_r_, I_geo_ (Cd, Hg—all three plots), RI, PLI (all three plots). Nevertheless, the cultivated agricultural crops do not show an increased accumulation of risk elements and in the case of Cd the contents were below the detection limit (Pb in spring triticale from Henckovce). However, the results indicate the necessity of soil monitoring, especially in risk areas, because by changing the conditions (pH, SOM), the release of bound forms of risk metals, which can subsequently enter the food chain.

## Data Availability

All basic data supporting the results of this study are available from the corresponding author.
